# Molecular detection of *bla*_CTX-M_ gene to predict phenotypic cephalosporin resistance and clinical outcome of *Escherichia coli* bloodstream infections in Vietnam

**DOI:** 10.1186/s12941-021-00466-3

**Published:** 2021-09-04

**Authors:** Trinh Van Son, Nguyen Dang Manh, Ngo Tat Trung, Dao Thanh Quyen, Christian G. Meyer, Nguyen Thi Kim Phuong, Phan Quoc Hoan, Vu Viet Sang, Dennis Nurjadi, Thirumalaisamy P. Velavan, Mai Hong Bang, Le Huu Song

**Affiliations:** 1 108 Institute of Clinical and Pharmaceutical Sciences, Hanoi, Vietnam; 2 Vietnamese-German Center for Medical Research (VG-CARE), Hanoi, Vietnam; 3 Institute of Clinical Infectious Diseases, 108 Military Central Hospital, Hanoi, Vietnam; 4 Institute of Tropical Medicine, Universitätsklinikum Tübingen, Tübingen, Germany; 5 Department of Microbiology, 108 Military Central Hospital, Hanoi, Vietnam; 6 Central Laboratory, 108 Military Central Hospital, Hanoi, Vietnam; 7 Department of Infectious Diseases, Heidelberg University Hospital, Heidelberg, Germany; 8 108 Military Central Hospital, Nr.1 Tran Hung Dao street, Hanoi, Vietnam

**Keywords:** Antimicrobial resistances, Blood stream infections, Sepsis, ESBL, AMR, CTX-M, Drug-resistant *Escherichia coli*

## Abstract

**Background:**

Blood stream infections (BSI) caused by Extended Spectrum Beta-Lactamases (ESBLs) producing *Enterobacteriaceae* is a clinical challenge leading to high mortality, especially in developing countries. In this study, we sought to describe the epidemiology of ESBL-producing *Escherichia coli* strains isolated from Vietnamese individuals with BSI, to investigate the concordance of genotypic-phenotypic resistance, and clinical outcome of ESBL *E. coli* BSI.

**Methods:**

A total of 459 hospitalized patients with BSI were screened between October 2014 and May 2016. 115 *E. coli* strains from 115 BSI patients were isolated and tested for antibiotic resistance using the VITEK®2 system. The ESBL phenotype was determined by double disk diffusion method following the guideline of Clinical and Laboratory Standards Institute. Screening for beta-lactamase (ESBL and carbapenemase) genes was performed using a multiplex-PCR assay.

**Results:**

58% (67/115) of the *E. coli* strains were ESBL-producers and all were susceptible to both imipenem and meropenem. Resistance to third-generation cephalosporin was common, 70% (81/115) were cefotaxime-resistant and 45% (52/115) were ceftazidime-resistant. *bla*_CTX-M_ was the most common ESBL gene detected (70%; 80/115) The sensitivity and specificity of *bla*_CTX-M_-detection to predict the ESBL phenotype was 87% (76–93% 95% CI) and 54% (39–48% 95% CI), respectively. 28%% (22/80) of *bla*_CTX-M_ were classified as non-ESBL producers by phenotypic testing for ESBL production. The detection of *bla*_CTX-M_ in ESBL-negative *E. coli* BSI was associated with fatal clinical outcome (27%; 6/22 versus 8%; 2/26, p = 0.07).

**Conclusion:**

A high prevalence of ESBL-producing *E. coli* isolates harbouring *bla*_CTX-M_ was observed in BSI patients in Vietnam. The genotypic detection of *bla*_CTX-M_ may have added benefit in optimizing and guiding empirical antibiotic therapy of *E. coli* BSI to improve clinical outcome.

**Supplementary Information:**

The online version contains supplementary material available at 10.1186/s12941-021-00466-3.

## Introduction

The emergence and spread of antimicrobial resistance (AMR) are of growing importance worldwide. With a population of more than 96 million and a high burden of infectious diseases, Vietnam faces since a decade a significant increase of AMR [1, 2]. Bloodstream infections (BSI) caused by multidrug-resistant bacteria frequently leads to fatal treatment failure [3, 4]. In Vietnam, BSIs are mostly caused by multidrug-resistant pathogens listed in the Global Antimicrobial Resistance Surveillance System (GLASS) report, including those classified as critical and high priority levels by the World Health Organization [5], such as extended spectrum beta-lactamase- (ESBL) and carbapenemase-resistant *Enterobacteriaceae* (CREs) [6, 7]. The most common cause of both community- and hospital-acquired BSI in South-East Asia is *Escherichia coli*, a member of the *Enterobacteriaceae* family [8, 9].

Wide dissemination of beta-lactamase producing *E. coli*, and increased incidences of hospital-acquired ESBL-producing *E. coli* infections have been documented [10–14]. In Italy, the incidences of community- and hospital-acquired infections by ESBL-producing *E. coli* increased between 2007 and 2015 from 29 to 42% and 25 to 35%, respectively [10]. Reports for the years 2002 until 2011 suggest an exponential increase in ESBL resistances from 20 to 45% in the Asia–Pacific region and from 39 to 55% in South-East Asia [15]. Most resistant bacterial strains were identified in patients treated at intensive care units (ICUs). In South-East Asia, Philippines and Vietnam have reported a high burden of ESBL-producing *E. coli* infections of 59% and 81%, respectively [16]. The most common resistance factors in clinical settings worldwide are the CTX-M-type beta-lactamases [17]. Furthermore, CRE carrying the transmissible carbapenemase genes *bla*_KPC_, *bla*_NDM_ and *bla*_OXA-48_ are well characterized across various geographical regions [18]. Local epidemiological data on AMR is essential in guiding empirical antibiotic therapies to improve clinical management and outcome of BSIs, while supporting antibiotic stewardship measures to combat the spread of AMR [9]. However, epidemiological data on AMR from low- and middle-income countries (LMIC) are scarce compared to industrialized nations with a well-functioning surveillance system, so that the AMR burden in LMIC may be underestimated.

The present study aims to describe the molecular epidemiology and resistance patterns of *E. coli* strains isolated from Vietnamese individuals with BSI using a retrospective study cohort. We also evaluated the concordance of genotypic and phenotypic resistance in *E. coli* causing BSI towards molecular detection of ESBL-genes to predict cephalosporin resistance in *E. coli* and to optimize the management of *E. coli* BSI in a high ESBL-prevalent setting.

## Materials and methods

### Patient recruitment

The study group included patients with BSI admitted to the 108 Military Central Hospital in Hanoi, Vietnam, between October 2014 and May 2016. The 108 Military Central Hospital is a hospital of maximum care for Northern Vietnam, providing medical care for patients in Hanoi and for those being referred from various regional hospitals. In the latter case patients´ previous antibiotic treatment scheme was provided by the referring hospital. 459 BSI patients with bacterial pathogens diagnosed by blood cultures were hospitalized in the 108 Military Central Hospital during the study period. Of those, 115 patients with blood cultures positive for *E. coli* were included in this study. Diagnoses of BSI followed the criteria of the Surviving Sepsis Campaign guidelines [19] and the sepsis-related organ failure assessment (SOFA) score.

### Blood culture

Two independent venous blood samples of approximately 8 mL per blood culture bottle were collected from both arms of the patients for blood culture with the BACTEC™ Plus Aerobic/F system (Becton–Dickinson, Franklin Lakes, NJ, USA). Blood culture bottles were incubated in the BD BACTEC™ 9120 device (Becton–Dickinson, Franklin Lakes, NJ, USA) at 36 °C for 18–72 h. Positive cultures were subjected to bacterial species identification and antimicrobial susceptibility testing using the VITEK® 2 compact automated system (BioMérieux, Lyon, France). Bacterial strains were stored with 20% glycerol at − 80 °C for further molecular testing.

### Antimicrobial susceptibility testing

The minimum inhibitory concentration (MIC) for a wide spectrum of antibiotics were tested using VITEK AST-N204 cards and the VITEK® 2 compact automated system (bioMérieux, Lyon, France). The interpretation of susceptibility patterns was done according to the Clinical and Laboratory Standards Institute (CLSI) guidelines [20]. The following antimicrobial substances were included: Ampicillin (AM), Amoxicillin/Clavulanic acid (AMC), Piperacillin/Tazobactam (TZP), Cefotaxime (CTX), Ceftazidime (CAZ), Cefepime (FEP), Ertapenem (ETP), Imipenem (IPM), Meropenem (MEM), Amikacin (AN) and Ciprofloxacin (CIP).

Phenotypic confirmation of ESBL production was achieved by double disk diffusion methods following the guideline of CLSI [20]. The CTX (30 µg) and CAZ (30 µg) disks alone and in combination with clavulanate (10 µg) were used for testing of ESBL phenotype *E. coli* on Mueller–Hinton agar. The tests were considered positive if the difference in the zone of clearance between CTX and CTX with clavulanate or CAZ and CAZ with clavulanate was ≥ 5 mm.

### Detection of beta-lactamases

Genes encoding ESBLs and carbapenemases were screened using multiplex PCR assays described previously [21]. Six genes encoding ESBL (*bla*_SHV_, *bla*_TEM_, *bla*_CTX-M_ [ESBL-1]; *bla*_VEB_, *bla*_GES_, *bla*_PER_ [ESBL-2]) and nine genes encoding carbapenamases (*bla*_NDM-1_, *bla*_SPM_, *bla*_VIM_ [CARBA-1]; *bla*_IMP_, *bla*_AIM_, *bla*_KPC_ [CARBA-2]; *bla*_OXA-23_, *bla*_OXA-48-like,_ *bla*_OXA-58_ [CARBA-3]) can be detected using the multiplex PCR panels (Additional file 1: Table S1). The primers were designed to detect the most common variants of the beta-lactamase genes irrespective of the potency of the beta-lactamase activity. Bacterial isolates were incubated at 35 ± 1 °C and colonies were picked and diluted in 300 μl TE buffer, followed by DNA extraction. In detail, the suspension was immersed in 300 μl universal lysis solution containing 200 mM NaOH and 1% SDS and incubated at 95 °C for 5 min. An equal volume of 1 M Tris–HCl was added to achieve a pH of 7.5. Subsequently, the solution was transferred to a 1.5 mL Eppendorf tube and an equal volume of phenol–chloroform-isoamyl alcohol mixture (25/24/1) (ThermoFisher Scientific, Invitrogen, Waltham, MA, USA) was added. The mixture was vortexed for 5 min and centrifuged at 13,200 rpm. The upper aqueous phase was transferred to an Eppendorf tube and an equal volume of isopropanol was added. After mixing, the solution was centrifuged at 13,200 rpm for 30 min to pellet the DNA. Precipitated DNA was washed twice with 70% ethanol and reconstituted in 150 μl TE buffer (25 mM Tris with 0.1 mM EDTA). Multiplex PCR assays were performed in 25 μl reaction volumes containing hot start master mix (2×) (Promega, San Luis Obispo, CA, USA) and individual primer pairs with varying concentrations (Additional file 1: Table S1). Thermal cycling conditions were denaturation at 95 °C for 2 min followed by 40 cycles of 94 °C for 30 s denaturation, 61 °C for 30 s annealing, followed by an extension at 72 °C for 40 s and a final extension step of 72 °C for 5 min. Amplicons were visualized on 1.2% agarose gels (Additional file 1: Figure S1).

### Statistical analysis

Statistical analyses were performed using the SPSS software v.23.0 (IBM Corporation, Chicago, IL, USA). Continuous variables are presented as mean ± standard deviation. Categorical variables are given as frequencies with percentages and comparisons of categorical variables between groups were performed using chi-square and Fisher’s exact tests. The level of significance was set at *p*-values < 0.05.

## Results

### Clinical characteristics of study subjects

Of the 115 patients, 70 (61%) were males. The mean age of the patients was 62 years; 62/115 patients were > 60 years old. In 73 patients (64%) pre-existing conditions (solid cancer, hypertension, diabetes, liver cirrhosis) were recorded and eight of them were receiving immunosuppressive therapy (Table 1). A primary source of infection was identified in 96 (84%) patients, with urine tract infection (40/96; 42%) and infection of the bile duct system (36/96; 38%) as the main sources, followed by respiratory and post interventional infections. The mean SOFA score was 3.36 ± 3.0 and the median procalcitonin level was 10.19 (0.24–100) ng/L. Shock and mortality rates were 17% and 16%, respectively (Table 1).

**Table 1:** Baseline characteristics of BSI patients with *Escherichia coli* infections

Baseline characteristics	Values as n (%)
Sex (male)	70/115 (61%)
Mean age in years	62.3 ± 16.2
≥ 60 years old	62(54%)
Pre-existing conditions	73/115 (64%)
Solid cancer	35/73 (48%)
Hypertension	33/73 (45%)
Diabetes	20/73 (27%)
Liver cirrhosis	14/73 (19%)
Immunosuppressive therapy	8/73 (11%)
Primary source infection n (%)	96/115 (84%)
Urine tract infection	40/96 (42%)
Bile duct infection	36/96 (38%)
Respiratory infection	8/96 (8%)
After intervention	5/96 (5%)
Others	7/96 (7%)
Mean SOFA (points)	3.36 ± 3.05
While Blood Cells (G/L)	16.9 ± 13.7
Neutrophile (G/L)	13.7 ± 9.9
Platelets (G/L)	190 ± 118
Prothrombin (%)	76.5 ± 22.8
Pro-Calcitonin median (range)(ng/L)	10.19 (0.24–100)
pH	7.33 ± 0.13
Lactate (mmol/L)	5.34 ± 3.53
Length of hospital stay (days)	19.9 ± 14.7
Shock n (%)	19/115 (17%)
Mortality n (%)	18/115 (16%)

### Phenotypic antimicrobial susceptibility

Of the 115 *E. coli* isolates, 67 (58%) produced ESBLs. The phenotypic resistances of all study isolates are summarized in Table 1. The most prevalent resistance to beta-lactam antibiotics was observed for AM (109/115; 95%), followed by CTX (81/115; 70%), CAZ (52/115; 45%), AMC (32/115; 28%), FEP (28/109; 26%) and TZP (8/115; 7%) (Table 2). Three *E. coli* strains exhibited reduced susceptibility to ETP; one of them resistant with a MIC value of 2 mg/L, and two with intermediate susceptibility with MIC values of 1 mg/L. All isolates were phenotypically susceptible to IPM and MEM. With regard to other antibiotics, 67% (77/115) of strains were resistant to CIP, but 99% (114/115) of strains were still susceptible to AN (Table 2).

**Table 2:** Phenotypic resistance of *Escherichia coli* causing BSI in Vietnam

	Overall, n = 115						ESBL^+^, n = 67						ESBL^−^, n = 48					
	S		I		R		S		I		R		S		I		R	
	n	%	n	%	n	%	n	%	n	%	n	%	n	%	n	%	n	**%**
AM	5	4.3	1	0.9	109	94.8	0	–	0	–	67	100.0	5	10.4	1	2.1	42	87.5
AMC	67	58.3	16	13.9	32	27.8	43	64.2	12	17.9	12	17.9	24	50.0	4	8.3	20	41.7
TZP	93	80.9	14	12.2	8	7.0	59	88.1	7	10.4	1	1.5	34	70.8	7	14.6	7	14.6
CAZ	62	53.9	1	0.9	52	45.2	31	46.3	1	1.5	35	52.2	31	64.6	0	–	17	35.4
CTX	34	29.6	0	0.0	81	70.4	0	0.0	0	0.0	67	100.0	34	70.8	0	–	14	29.2
FEP^a^	68	62.4	13	11.9	28	25.7	34	55.7	11	18.0	16	26.2	34	70.8	2	4.2	12	25.0
ETP	112	97.4	2	1.7	1	0.9	66	98.5	1	1.5	0	–	46	95.8	1	2.1	1	2.1
IPM	115	100.0	0	–	0	–	67	100.0	0	–	0	–	48	100.0	0	–	0	–
MEM	115	100.0	0	–	0	–	67	100.0	0	–	0	–	48	100.0	0	–	0	–
AN	113	98.3	1	0.9	1	0.9	67	100.0	0	–	0	–	46	95.8	1	2.1	1	2.1
GM	73	63.5	3	2.6	39	33.9	43	64.2	1	1.5	23	34.3	30	62.5	2	4.2	16	33.3
CIP	37	32.2	1	0.9	77	67.0	17	25.4	1	1.5	49	73.1	20	41.7	0	–	28	58.3

AMC: Amoxicillin/Clavulanic acid; TZP: Piperacillin/Tazobactam; ESBL: extended spectrum beta lactamase; CTX: Cefotaxime; CAZ: Ceftazidime; FEP: Cefepime; AN: Amikacin; CIP: Ciprofloxacin; S: susceptible; I: intermediate; R: resistant

^a^missing values for FEP susceptibility, n = 6 in ESBL-producers (ESBL^+^)

### Detection of beta-lactamases

The proportion of strains carrying *bla*_CTX-M_, *bla*_TEM_, *bla*_SHV_ was 70% (80/115), 58% (67/115) and 1% (1/115), respectively. Of the 115 strains, 105 (91%) carried at least one of the three resistance genes (*bla*_CTX-M_, *bla*_CTX-M_, *bla*_SHV_); none of the strains carried all three genes. The combination of *bla*_CTX-M_ and *bla*_TEM_ was detected in 42 (37%) of the 115 strains (Table 3). 9/115 (8%) strains were positive for *bla*_NDM_ and 5/115 (4%) carried the *bla*_VIM_ gene.

**Table 3:** Concordance of genotypic and phenotypic resistance in *bla*_CTX-M_-harbouring *Escherichia coli*

Antibiotic substance^b^	*bla*_CTX-M_, n = 80^a^						*bla*_CTX-M_-positive ESBL-producers, n = 58^a^						*bla*_CTX-M_-positive non ESBL producers, n = 22^a^					
	S		I		R		S		I		R		S		I		R	
	n	%	n	%	n	%	n	%	n	%	n	%	n	%	n	%	n	%
AM	0	–	0	–	80	100	0	–	0	–	58	100	0	–	0	–	22	100
AMC	48	60	9	11.3	23	28.8	40	69	9	15.5	9	15.5	8	36.4	0	–	14	63.6
TZP	64	80	9	11.3	7	8.8	51	87.9	6	10.3	1	1.7	13	59.1	3	13.6	6	27.3
CAZ	35	43.8	1	1.3	44	55	27	46.6	1	1.7	30	51.7	8	36.4	0	–	14	63.6
CTX	11	13.8	0	–	69	86.3	0	–	0	–	58	100	11	50	0	–	11	50
FEP^c^	37	50	10	13.5	27	36.5	29	55.8	8	15.4	15	28.8	8	36.4	2	9.1	12	54.5
ETP	77	96.3	2	2.5	1	1.3	57	98.3	1	1.7	0	–	20	90.9	1	4.5	1	4.5
IPM	80	100	0	–	0	–	58	100	0	–	0	–	22	100	0	–	0	–
MEM	80	100	0	–	0	–	58	100	0	–	0	–	22	100	0	–	0	–
AN	78	97.5	1	1.3	1	1.3	58	100	0	–	0	–	20	90.9	1	4.5	1	4.5
GM	52	65	3	3.8	25	31.3	39	67.2	1	1.7	18	31	13	59.1	2	9.1	7	31.8
CIP	18	22.5	1	1.3	61	76.3	14	24.1	1	1.7	43	74.1	4	18.2	0	–	18	81.8

AMC: Amoxicillin/Clavulanic acid; TZP: Piperacillin/Tazobactam; ESBL: extended spectrum beta lactamase; CTX: Cefotaxime; CAZ: Ceftazidime; FEP: Cefepime; AN: Amikacin; CIP: Ciprofloxacin; S: susceptible; I: intermediate; R: resistant

^a^May not add up to 100% due to rounding errors to 1 decimal place

^b^Interpretation of resistance according to the clinical breakpoints of CLSI

^c^6 missing values for cefepime

The *bla*_NDM_ gene was detected in 2 isolates carrying the *bla*_CTX-M_ gene, in 2 isolates carrying the *bla*_TEM_, and in 5 isolates harbouring both *bla*_CTX-M_ and *bla*_TEM_ genes. The *bla*_VIM_ gene was detected in 4 isolates harbouring the *bla*_CTX-M_, and in one isolate harboring both *bla*_CTX-M_ and *bla*_TEM_ genes.We did not detect strains carrying *bla*_KPC_, *bla*_VEB_, *bla*_PER_, *bla*_GES_, *bla*_IMP_, *bla*_SMP_, *bla*_AIM_, *bla*_OXA-23_, *bla*_OXA-48-like_ and *bla*_OXA-58_.

### Concordance of resistance genotypes and phenotypic susceptibility

The concordance of genotypic and phenotypic resistance is summarized in Table 3. 14 out of 81 phenotypically CTX-resistant isolates were non-ESBL-producers according to the double disk method. However, 5 out of the 14 harboured *bla*_CTX-M_, 2 harboured *bla*_TEM_, 6 harboured both *bla*_CTX-M_ and *bla*_TEM_, and one isolate did not harbour any beta-lactamase genes in our test-panel. There was a discrepancy between the detection of *bla*_CTX-M_ and the phenotypic testing for ESBL, 22 out of 80 (27.5%) of isolates with *bla*_CTX-M_ were classified as non-ESBL-producers by the double disk method. In our study setting, the sensitivity and specificity of *bla*_CTX-M_ detection to predict the ESBL phenotype was 87% (95% CI 76–93%) and 54% (95% CI 39–68), respectively. A summary of the sensitivities and specificity of *bla*_CTX-M_ detection to predict phenotypic resistance to CAZ, CTX and FEP are summarized in Table 4.

**Table 4:** Performance of *bla*_CTX-M_ detection to predict phenotypic resistance of *Escherichia coli*

Antibiotic	Sensitivity % (95% CI)	Specificity % (95% CI)	PPV % (95% CI)	NPV % (95% CI)
ESBL	87 (76–93)	54 (39–68)	73 (61–82)	74 (56–87)
CTX	85 (75–92)	68 (49–82)	86 (76–93)	66 (48–80)
CAZ	85 (72–93)	44 (31–57)	57 (45–67)	77 (59–89)
FEP	90 (76–97)	46 (34–58)	50 (38–62)	89 (72–96)

ESBL: Extended spectrum beta lactamases; CTX: Cefotaxime; CAZ: Ceftazidime; FEP: Cefepime; and CIP: Ciprofloxacin; PPV: positive predictive value; NPV: negative predictive value; 95% CI: 95% confidence interval

Strikingly, all isolates carrying *bla*_NDM_ and *bla*_VIM_ were phenotypically susceptible to all carbapenems (ETP, IPM and MEM), which was unusual. The prevalence of quinolone resistance was overall very high in our isolate collection. However, there was an over-representation of quinolone resistance in *bla*_CTX-M_-carrying isolates.

### *bla*_CTX-M_ in cephalosporin-susceptible *E. coli* associated with unfavorable clinical outcome

**Fig. 1:**
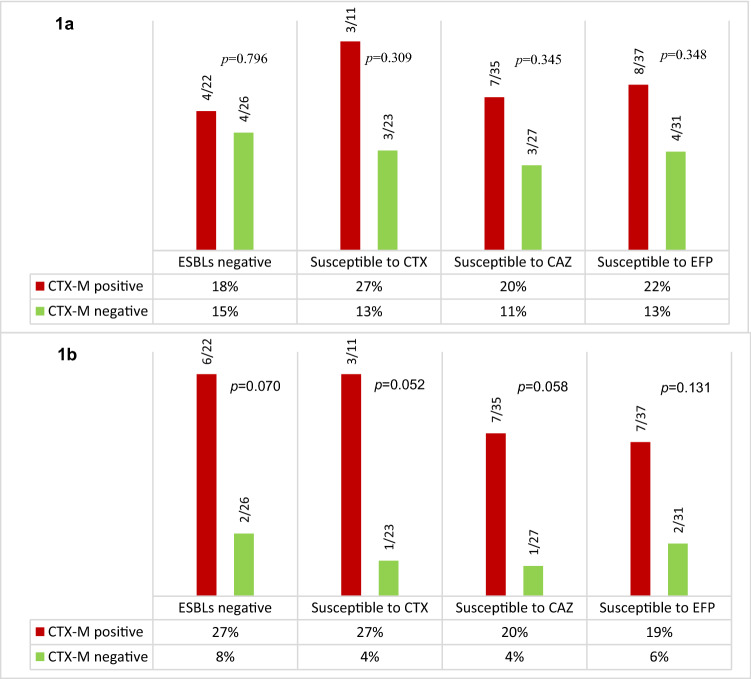
The contribution of *bla*_CTX-M_ to the outcome of BSI patients caused by cephalosporin susceptible *Escherichia coli*. **a** For ratio of shock and **b** for fatality. Red column is ratio of shock (**a**) or death (**b**) of BSI patients caused by *bla*_CTX-M_ positive *E. coli* and green column is ratio of shock (**a**) or death (**b**) of BSI patients caused by *bla*_CTX-M_ negative *E. coli*

Since not all *bla*_CTX-M_-habouring isolates exhibited phenotypic resistance to third- and fourth-generation cephalosporins tested, we performed an analysis comparing the clinical outcome (shock and fatality) of BSI with cephalosporin-susceptible *E. coli* with *bla*_CTX-M_ as an exposure variable. Our analysis suggested that patients with *bla*_CTX-M_-negative *E. coli* BSI had better clinical outcomes than patients with *bla*_CTX-M_-positive *E. coli* BSI. In particular, the presence of *bla*_CTX-M_ in phenotypically cephalosporin-susceptible *E. coli* BSI is associated with fatal outcome of the infection for CTX-susceptible phenotype (4% versus 27%, p = 0.05), and with a statistical trend for ESBL-negative phenotype (8% versus 27%, p = 0.07), for CAZ-susceptible phenotype (4% versus 20%, p = 0.06) for ESBL-negative phenotype (4% versus 27%, p = 0.05), for FEP-susceptible phenotype (6 versus 19%, p = 0.1) (Fig. 1, Additional file 1: Table S2). In addition, the duration of hospital stay was significantly longer in patients with CTX-M-positive *E. coli* infections than in patients with CTX-M-negative *E. coli* BSI (Additional file 1: Table S2). For discrepant genotypic-phenotypic carbapenemase susceptibility, there was no association between the discrepancy and the clinical outcome (neither shock nor death). This analysis was performed with very small number of cases so that further investigations are needed before drawing any definitive conclusions.

## Discussion

In this study, we reported a high prevalence (58%) of ESBL-producers in *E. coli* causing BSI in Vietnamese patients. Our findings are in accordance with previously published data on the epidemiology of ESBL-producing *E. coli* from Vietnam, which suggested a comparable (48–55%) prevalence [9, 16, 22]. Similar findings have been reported from studies conducted in in China (56–67%) [22, 23] and other regions in South-East Asia, such as Thailand (50%) [22]. In comparison, studies from industrialized (middle to high income) countries reported lower rates of ESBL-producers in clinical *E. coli* strains. A study from Canada has reported a far lower rate of 4% ESBL-producing *E. coli* strains [24]. Another study conducted in Sweden reported similar low rates of ESBL-producing *E. coli* strains with 2%, 3% and 4% of cases in 2007, 2009 and 2011, respectively [25]. Taken together, these discrepancies illustrate and highlight the increasing AMR problematic in the Asian region and in LMIC, in general. Surveillance, antibiotic use and higher compliance with antibiotic stewardship might account for these differences [26]. In many South-East Asian countries including Vietnam, antibiotics, especially of broad-spectrum cephalosporins can be purchased over the counter without prescription. Thus, inappropriate indications for antibiotic therapy may have an influence on the high rates of resistance [27, 28].

Almost all (97%) ESBL-producing *E. coli* in our study harboured at least one beta-lactamase gene, with *bla*_CTX-M and_ *bla*_TEM_ as the most common, consistent with published studies from Thailand and Vietnam [29, 30]. Indeed, among the 300 ESBL subtypes [31], the TEM-, SHV- and CTX-M-types are predominant and of particular relevance [32]. The CTX-M-type beta-lactamases play a major role as emerging and the most widely spread resistance factors in *Enterobacteriaceae* on a global scale [17]. Strikingly, we found a significant proportion (46%) of non ESBL-producers, as determined by phenotypic testing for ESBL, carrying the *bla*_CTX-M_ gene. Since our multiplex PCR was designed to detect the most common CTX-M types, we cannot rule out that this discrepancy is attributed to CTX-M subtypes with weaker beta-lactamase activity. Overall, the concordance between phenotypic and genotypic susceptibility was good for *bla*_CTX-M_-carrying isolates. However, the presence of *bla*_CTX-M_ alone cannot predict phenotypic resistance to cephalosporins perfectly. Not all CTX-M subtypes are equally potent; depending on the expression, the beta-lactamase activity levels may be variable [17, 33]. Furthermore, the presence of insertion sequences, such as IS*E cp1B* and IS*903D*, can modify the expression of the ESBL genes [34]. This highlights the necessity of implementing high-resolution molecular typing methods, such as whole-genome sequencing, to study the molecular epidemiology of AMR and multidrug-resistant pathogens in high-prevalent settings.

Carbapenem-resistance is low in *E. coli* causing BSI in our study population. Our study shows 98% ETP and 100% IPM and MEM susceptibility of *E. coli* isolates. However, we detected the presence of the Ambler class B metallo-beta-lactamase in 12 isolates, 8 with *bla*_NDM_ and 4 with *bla*_VIM_. Oddly, the presence of these genes did not correlate with the phenotypic resistance to any carbapenems tested, which we could not explain. Indeed, discrepancies between genotypic und phenotypic resistances have been described previously [35]. The primers for both *bla*_NDM_ and *bla*_VIM_ were not specific for a particular variant; therefore, it is possible that nucleotide variations leading to a non-functional protein cannot be ruled out. In addition, the expression of beta-lactamase genes may be affected by promoter regions or other genetic element of the plasmid carrying these AMR genes [36]. Nonetheless, the high susceptibility rates for carbapenems and TZP indicate that these substances may be appropriate for the empirical antibiotic therapy of *E. coli* BSI in Vietnam. Of note, CIP resistance rate of 67% can be considered high in comparison to other studies [37], which may have been the result of frequent use of CIP.

The molecular detection of *bla*_CTX-M_ in non-ESBL and cephalosporin-susceptible *E. coli* was significantly associated with a worse clinical outcome (septic shock and fatality) of *E. coli* BSI. This finding was unexpected since phenotypic susceptibility is considered as the gold standard for guiding antimicrobial therapies. The increased expression of antimicrobial resistance genes under antibiotic pressure may be an explanation for this observation [38]. Exposure to an antimicrobial substance may induce stress response (SOS-response) in bacteria, which causes several genes including AMR genes, such as *bla*_CTX-M_ to be up-regulated thus increasing tolerance to various substance classes (cross-resistance) [39, 40]. In such cases, antibiotic therapy guided by phenotypic resistance only may not be suitable (i.e., false susceptible) and, thus, may lead to a poor outcome as a consequence of undertreatment. In this study cohort, we did not observe any significant differences in the clinical outcome for cases with discrepant phenotypic and genotypic carbapenem susceptibility. Due to the small number of cases (n = 14), we would refrain from drawing any conclusions. Nonetheless, this finding is unexpected and warrants further scrutiny. The time to antibiotic therapy initiation may influence the outcome of an infection. Therefore, the acceleration of time to report is of particular interest in the routine microbiological diagnostics [41]. Conventional blood culture diagnostics often take more than 24–48 h until antibiotic susceptibility profiles are available for the attending physicians, the introduction of genotypic PCR-based AMR detection may accelerate this process and thus potentially increasing the accuracy of calculated empirical antimicrobial therapy [42]. However, further validation in a larger cohort is needed prior to implementation in the routine setting.

Our study has limitations, the small sample size, especially for the clinical outcome analysis, may limit the generalizability of our findings. Nonetheless, with our small cohort, we could show that the presence of *bla*_CTX-M_ in cephalosporin-susceptible *E. coli* correlated with a poor outcome and warrant further investigations. Furthermore, our PCR panel encompasses multiple variants of the beta-lactamase genes. The primers were designed to detect the most common variants of the respective beta-lactamase genes, which may have resulted in discordance in the genotypic-phenotypic susceptibility for low-activity beta-lactamases, such as *bla*_TEM-1_, *bla*_TEM-2_ and *bla*_SHV-1_ [43]. Due to this limitation, the study focused on *bla*_CTX-M_.

Taken together, our data contributes to epidemiological data on the AMR burden in South-East Asia, which needs attention and should be closely monitored. The detection of AMR gene does not necessarily correlate with the phenotypic resistance in *E. coli* and warrant further scrutiny. Easy to implement PCR-based AMR detection method may have added benefit, especially in high-prevalent settings, to accelerate, optimize and guide antimicrobial therapy. The surveillance of the spread of multidrug resistance in LMIC is still suboptimal and access to high-resolution molecular typing methods may help combat the spread of AMR genes on a global scale.

## Supplementary Information

**Additional file 1: Table S1.** Primer sequences used for screening of beta-lactamase encoding genes. **Table S2.** Duration of hospital stay of patients with BSIs caused by cephalosporin susceptible *E. coli.* **Figure S1:** Agarose gel electrophoresis of *bla*_CTX-M_ (739 bp), *bla*_CTX-M_ (590 bp), *bla*_TEM_ (422 bp) genes; M50 Marker (50–1000 bp); (–) negative control; (+) positive control. Samples 1,2,3,5,6,9,13 are positive for *bla*_TEM_ and *bla*_CTX-M_; samples 4,11,12 are positive for *bla*_CTX-M_; samples 7 and 10 are negative for all (*bla*_SHV_, *bla*_CTX-M_ and *bla*_TEM_)

## Data Availability

All data are available within this manuscript.
