# Supplementary material for: Molecular detection of blaCTX-M gene to predict phenotypic cephalosporin resistance and clinical outcome of Escherichia coli bloodstream infections in Vietnam
Source: Ann Clin Microbiol Antimicrob. 2021 Sep 4;20:60. doi: 10.1186/s12941-021-00466-3 (PMC8418716; doi:10.1186/s12941-021-00466-3)
Supplement: Supplementary file 1 — Additional file 1: Table S1. Primer sequences used for screening of beta-lactamase encoding genes. Table S2. Duration of hospital stay of patients with BSIs caused by cephalosporin susceptible E. coli. Figure S1: Agarose gel electrophoresis of blaCTX-M (739 bp), blaCTX-M (590 bp), blaTEM (422 bp) genes; M50 Marker (50–1000 bp); (–) negative control; (+) positive control. Samples 1,2,3,5,6,9,13 are positive for blaTEM and blaCTX-M; samples 4,11,12 are positive for blaCTX-M; samples 7 and 10 are negative for all (blaSHV, blaCTX-M and blaTEM) [file 12941_2021_466_MOESM1_ESM.docx]

**Additional file**

**Table S1. Primer sequences used for screening of beta-lactamase encoding genes.**

| **Target** | **Target gene(s)** | **Primer sequences** (5’-3’) | **Product size** (bp) |
| --- | --- | --- | --- |
| ***ESBL-1*** | CTX-M-F | ATGTGCAGYACCAGTAARGTKATGGC | 590 |
|  | CTX-M-R | GGTRAARTARGTSACCAGAAYCAGCGG |  |
|  | TEM-F | TCGCCGCATACACTATTCTCAGAATGAC | 422 |
|  | TEM-R | CAGCAATAAACCAGCCAGCCGGAAG |  |
|  | SHV-F | TGTATTATCTC(C/T)CTGTTAGCC(A/G)CCCTG | 739 |
|  | SHV-R | GCTCTGCTTTGTTATTCGGGCCAAGC |  |
|  |  |  |  |
| ***ESBL-2*** | VEB-F | GATGGTGTTTGGTCGCATATCGCAAC | 391 |
|  | VEB-R | CATCGCTGTTGGGGTTGCCCAATTTT |  |
|  | PER-F | CAGTGTGGGGGCCTGACGAT | 731 |
|  | PER-R | CTGAGCA ACC TGC GCA ATR ATA GCT T |  |
|  | GES-F | CTGGCAGGGATCGCTCACTC | 604 |
|  | GES-R | GGTTTCCGATCAGCCACCTCTCA |  |
|  |  |  |  |
| ***CARBA-1*** | VIM-F | GATGGTGTTTGGTCGCATATCGCAAC | 390 |
|  | VIM-R | CGAATGCGCAGCACCAGGATAGAA |  |
|  | SPM-F | CGTTTGAAAATCTGGGTACGCAAACG | 291 |
|  | SPM-R | GTTTCAAATCAAAAACATTATCCGCTGGAACAG |  |
|  | NDM-1-F | CGAAAGTCAGGCTGTGTTGCGC | 200 |
|  | NDM-1-R | GACCGCCCAGATCCTCAACTG |  |
|  |  |  |  |
| ***CARBA-2*** | KPC-F | GCTTTCT(T/G)GCTG(C/G)CGC(T/C)GTGCT | 412 |
|  | KPC-R | AGCCAATCAAC(A/C)A(A/G)CTGCTG(C/A)CGC |  |
|  | AIM-F | CCCTGAAGGTGTACGGAAACAC | 326 |
|  | AIM-R | GGGTTCGGCCACCTCGAATTG |  |
|  | IMP-F | AC(G/A)GG(C/G/T)GGAATAGAGTGGCTTAA(T/C)TCTC | 204 |
|  | IMP-R | TTCAGG(C/T)A(A/G)CCAAACYACTASGTTATCT |  |
|  |  |  |  |
| ***CARBA-3*** | OXA-58-F | CCCCTCTGCGCTCTACATACAACATC | 599 |
|  | OXA-58-R | AAGTATTGGGGCTTGTGCTGAGCATAG |  |
|  | OXA-23-F | AGAATATGT(G/C)CC(A/T)GC(C/A)TC(T/A)ACATTTAA(A/G)ATG | 491 |
|  | OXA-23-R | CCCA(G/A)CC(G/T)GT(C/T)AACCA(G/A)CC |  |
|  | OXA-48-F | CACCAAGTCTTTAAGTGGGATGGACA | 300 |
|  | OXA-48-R | CCGATACGTGTAACTTATTGTGATACAGCTT |  |

**Table S2. Duration of hospital stay of patients with BSIs caused by cephalosporin susceptible *E. coli***

| ***E.coli* in BSI patients** | **Length of hospital stay (**days**)**  (Mean ± SD (n)) | | |
| --- | --- | --- | --- |
|  | ***bla*_CTX-M_ *(+)*** | ***bla*_CTX-M_ *(-)*** | ***p*** |
| **BSI patients with *E. coli susceptible to* cephalosporin** | | | |
| **ESBL *negative*** | 27.4±24.5 (n=22) | 14.4±7.5 (n=26) | 0.014 |
| ***Susceptible to CTX*** | 20.8±12.6 (n=11) | 12.9±5.9 (n=23) | 0.017 |
| ***Susceptible to CAZ*** | 17.8±9.3 (n=35) | 13.0±5.6 (n=27) | 0.023 |
| ***Susceptible to FEP*** | 17.2±8.5 (n=37) | 14.6±7.1 (n=31) | 0.185 |
| **Survival of BSI patients with *E. coli susceptible to* cephalosporin** | | | |
| **ESBL *negative*** | 28.3±16.2 (n=16) | 14.5±7.0 (n=24) | 0.001 |
| ***Susceptible to CTX*** | 24.1±12.2 (n=8) | 13.4±5.5 (n=22) | 0.002 |
| ***Susceptible to CAZ*** | 19.3±8.8 (n=28) | 13.5±5.3 (n=26) | 0.005 |
| ***Susceptible to FEP*** | 18.8±7.9 (n=30) | 14.7±6.7 (n=29) | 0.035 |

**Note:** Extended spectrum beta lactamases (ESBL), Cefotaxime (CTX), Ceftazidime (CAZ), Cefepime (FEP).

**Figure S1:** Agarose gel electrophoresis of *bla*_CTX-M_  (739 bp), *bla*_CTX-M_ (590bp), *bla*_TEM_ (422 bp) genes; M50 Marker (50-1000bp); (-) negative control; (+) positive control. Samples 1,2,3,5,6,9,13 are positive for *bla*_TEM_ and *bla*_CTX-M_; samples 4,11,12 are positive for *bla*_CTX-M_; samples 7 and 10 are negative for all (*bla*_SHV_, *bla*_CTX-M_ and *bla*_TEM_)

**
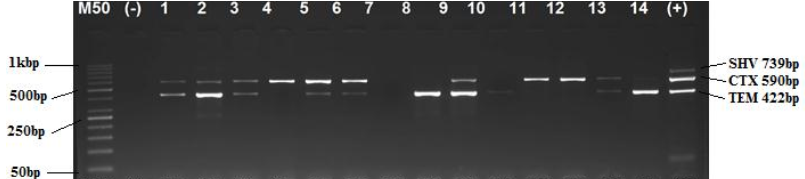
**
